# Neutral zone or conventional implant-retained overdentures? an assessment on quality of life, denture stability, patient satisfaction and maintenance requirements

**DOI:** 10.1186/s12903-025-06207-8

**Published:** 2025-05-26

**Authors:** Buse Cebi Gul, Murat Doğuş Günel, Tan Fırat Eyüboğlu, Mutlu Özcan

**Affiliations:** 1 Department of Prosthodontics, Faculty of Dentistry, Inonu University, Malatya, 44280 Turkey; 2 Department of Biostatistics, Faculty of Medicine of Ankara University, Ankara, Turkey; 3 Faculty of Dentistry, Department of Endodontics, Medipol University, Istanbul, Turkey; 4 Center for Dental Medicine, University of Zurich, Clinic of Masticatory Disorders and Dental Biomaterials, Zurich, Switzerland

**Keywords:** Edentulism, Implant retained overdentures, Neutral zone technique, OHIP EDENT, Oral health–related quality of life, Patient satisfaction, Prosthodontics

## Abstract

**Purpose:**

This study evaluated whether implant-retained overdentures fabricated using the neutral zone (NZ) technique offer superior long-term oral health related quality of life (OHRQoL) outcomes compared to conventional overdentures (CO) and assess the influence of these techniques on denture stability, patient satisfaction, and maintenance requirements.

**Materials and methods:**

This study involved 29 edentulous patients (14 in the NZ group and 15 in the CO group) who received mandibular implant-retained overdentures with a minimum two year follow up. Two implants were placed in the mandible for each patient, and overdentures were fabricated using either conventional methods or the neutral zone concept, all performed by the same clinician and technician. The OHIP-EDENT questionnaire assessed functional, physical, psychological, and social aspects. Statistical analyses compared pre and post treatment scores as well as intergroup differences.

**Results:**

Both groups demonstrated significant post-treatment improvement in OHRQoL (*p* < 0.001). However, no statistically significant difference was observed between the NZ and conventional groups in overall satisfaction, functional outcomes, pain reduction, or psychological and social well-being (*p* > 0.05). Furthermore, no significant difference was found in prosthesis fracture rates or the frequency of locator replacements (*p* > 0.05).

**Conclusions:**

Implant-retained overdentures, regardless of fabrication technique, significantly improved patient reported outcomes. However, the NZ technique did not provide a significant advantage over the conventional technique in long term OHRQoL or prosthodontic maintenance.

## Introduction

Edentulism is a common condition among older adults [1]. Projections on a global scale suggest that by 2050, the population aged 65 and older will be more than twice the number of children under five and will also exceed the demographic of individuals aged 15 to 24 [2]. Furthermore, it is estimated that 7–69% of adults worldwide may experience complete edentulism [3].

Implant supported complete dentures are an effective treatment option, providing dental clinicians with a way to enhance patients’ oral health and overall well-being [1]. In accordance with the McGill and York consensus statements, although overdentures supported by two implants are not universally considered the absolute “gold standard” in implant-based therapy, they do serve as a key benchmark. This standard is generally sufficient for most individuals, considering factors such as functional efficiency, patient acceptance, financial feasibility, and the clinical time involved [4].

The neutral zone (NZ) is defined as the specific area within the potential denture space where the oral musculature will not dislodge the prosthesis during function. It is regarded as an important factor for denture stability. Although high level evidence from well-controlled clinical trials is lacking, experts agree that the NZ concept should be adhered to during the fabrication of conventional complete dentures [5]. From the few published studies, it appears that NZ dentures may have certain clinical advantages over conventional dentures [6, 7, 8]. There are also a few studies examining how conventional and NZ dentures affect quality of life. In addition, one study investigates the effect of the neutral zone technique on bone loss around implants [9]. However, no study to date has directly compared how the conventional versus NZ technique in overdentures might differentially affect oral health related quality of life (OHRQoL).

The primary goal of rehabilitating individuals without natural teeth is to improve their oral health related quality of life (OHRQoL). Monitoring therapeutic success through patient-centered measures (PBOs) is essential [10]. OHRQoL scales, such as the Oral Health Impact Profile (OHIP) developed by Locker, assess the influence of dental care on oral health and overall well being [11, 12]. The OHIP includes 49 items across seven categories: functional limitation, physical pain, psychological discomfort, physical disability, psychological disability, social disability, and handicap. Despite its reliability and validity, the full OHIP can be lengthy and complex for patients to fully comprehend [13, 14].

In response to these limitations, Slade developed the OHIP-14, a concise 14-item version of the original OHIP that retains its seven domains and demonstrates acceptable validity, requiring less time for researchers and being easier for participants [13]. However, it may not fully suit individuals with complete dentures (CD), potentially causing a floor effect in measuring post-intervention improvements [14]. To address this, Allen and Locker [13] proposed the OHIP-EDENT, a 19-item profile specifically for edentulous patients, effectively capturing changes in oral health-related quality of life (OHRQoL) before and after new CD placement, which aids in developing targeted interventions [15].

Therefore, this study aimed to evaluate long-term oral health-related quality of life using the OHIP-EDENT in two different implant-retained complete denture fabrication techniques one applying the neutral zone concept and one not in patients with edentulism who presented to our clinic. We also investigated whether the neutral zone technique could positively impact the frequent need to replace or repair worn-out attachment inserts (locators), reducing overall maintenance costs. This study hypothesizes that implant retained complete dentures created using the neutral zone (NZ) technique will significantly enhance oral health related quality of life (OHRQoL) as measured by the OHIP-EDENT, compared to traditional implant retained complete dentures in edentulous patients. Furthermore, the NZ technique is anticipated to decrease the frequency of replacement or repair of worn attachment inserts (locators), thus reducing overall maintenance costs.

## Materials and methods

This investigation involved reviewing the records of overdenture patients treated at the university dental clinic from January 2018 to December 2022. Patients who had received overdentures in the preceding two years were excluded to ensure a minimum follow-up period of 24 months. The study was approved by the Non-Interventional Ethical Committee of Inonu University (Approval No: 2025/7035). Informed consent forms regarding the content of the study were obtained from all participants. A paired design was used to test whether the paired difference in distributions (δ) differed from zero (H₀: δ = 0, H_1_: δ ≠ 0). Comparisons were performed using a two-sided Wilcoxon Signed-Rank test for paired differences, with a Type I error rate (α) of 0.05. A sample size of 25 was calculated to detect a difference in satisfaction scores before and after treatment with an effect size of 0.6 and 80% power.

Participants who needed a complete denture and were set to receive an implant retained complete denture were enrolled in the study. Two cohorts were compared: one group received an implant retained complete denture made using the neutral zone technique, while the other did not. Both groups underwent a long term satisfaction assessment utilizing the Oral Health Impact Profile for edentulous subjects (OHIP-EDENT) [13, 15]. The inclusion criteria required the absence of any systemic condition affecting bone resorption or implant osseointegration, no hearing or speech impairment, a normal tongue size and function, and the ability and willingness to understand and complete the satisfaction questionnaire. Patients who did not meet these criteria were excluded from the study.

The same clinician performed all clinical procedures, while all laboratory processes took place in a single facility with the same technician. Conventional dentures followed the standard protocol adopted at the dental school. Preliminary impressions were made using an irreversible hydrocolloid material (Cavex CA37; Cavex Holland BV) in stock trays adjusted as needed. Border molding employed impression compound (Kerr Corp), and a zinc oxide–eugenol material (Cavex Outline) was subsequently used to obtain the final impression in custom trays fashioned from cold-cured acrylic resin (Acrostone). The definitive impressions were boxed and poured in dental stone to generate master casts. The maxillary wax rims were adjusted to achieve proper lip support and accurately orient the occlusal plane. The occlusal vertical dimension was set to 3 mm below the resting vertical dimension, and the mandibular cast was subsequently mounted. Artificial teeth (Vertex Quint Teeth; Vertex Dental) were positioned according to a bilateral balanced occlusal arrangement, initially placing the mandibular teeth along the ridge crest. Clinical procedures continued with a try-in, denture insertion, and a follow-up visit one week later.

**Fig. 1:**
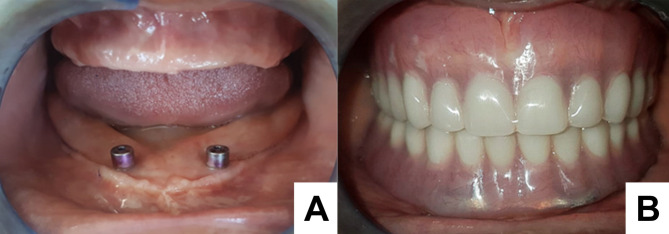
The positioning of maxillary teeth according to a bilaterally balanced occlusal scheme, consistent with standard denture protocols

Fabrication of the NZ denture mirrored the conventional approach up to the point of occlusal registration and articulator mounting. The mandibular wax rim and trial denture base were replaced at that juncture with an acrylic resin plate incorporating a wire spanning the posterior wax segments. Intraoral acrylic resin stops were formed to reflect the previously determined occlusal vertical dimension. The NZ impression involved applying a tissue-conditioning agent (Visco-gel; Dentsply Sirona) [16, 17] onto both the resin plate and the wire structure, contouring it to resemble the shape of the original wax rim. Next, the participant was seated upright with the maxillary occlusal rim in position and asked to perform the following actions over a 10-minute interval: gentle swallowing, frequent sipping of water, speaking aloud, pronouncing vowels, counting from 60 to 70, smiling, grimacing, licking the lips, and pursing the lips. Subsequently, the NZ record was boxed using a putty silicone material (Labor Plus; Dental Line LTD) [16, 17] to obtain both lingual and facial matrices. The tissue-conditioning material was then substituted with wax, guided by these indices to accurately replicate the NZ record. Then, the mandibular artificial teeth were arranged, and the flanges were contoured following the indices. Finally, the maxillary teeth were positioned according to a bilaterally balanced occlusal scheme, consistent with standard denture protocols (Fig. 1A and B).

**Fig. 2:**
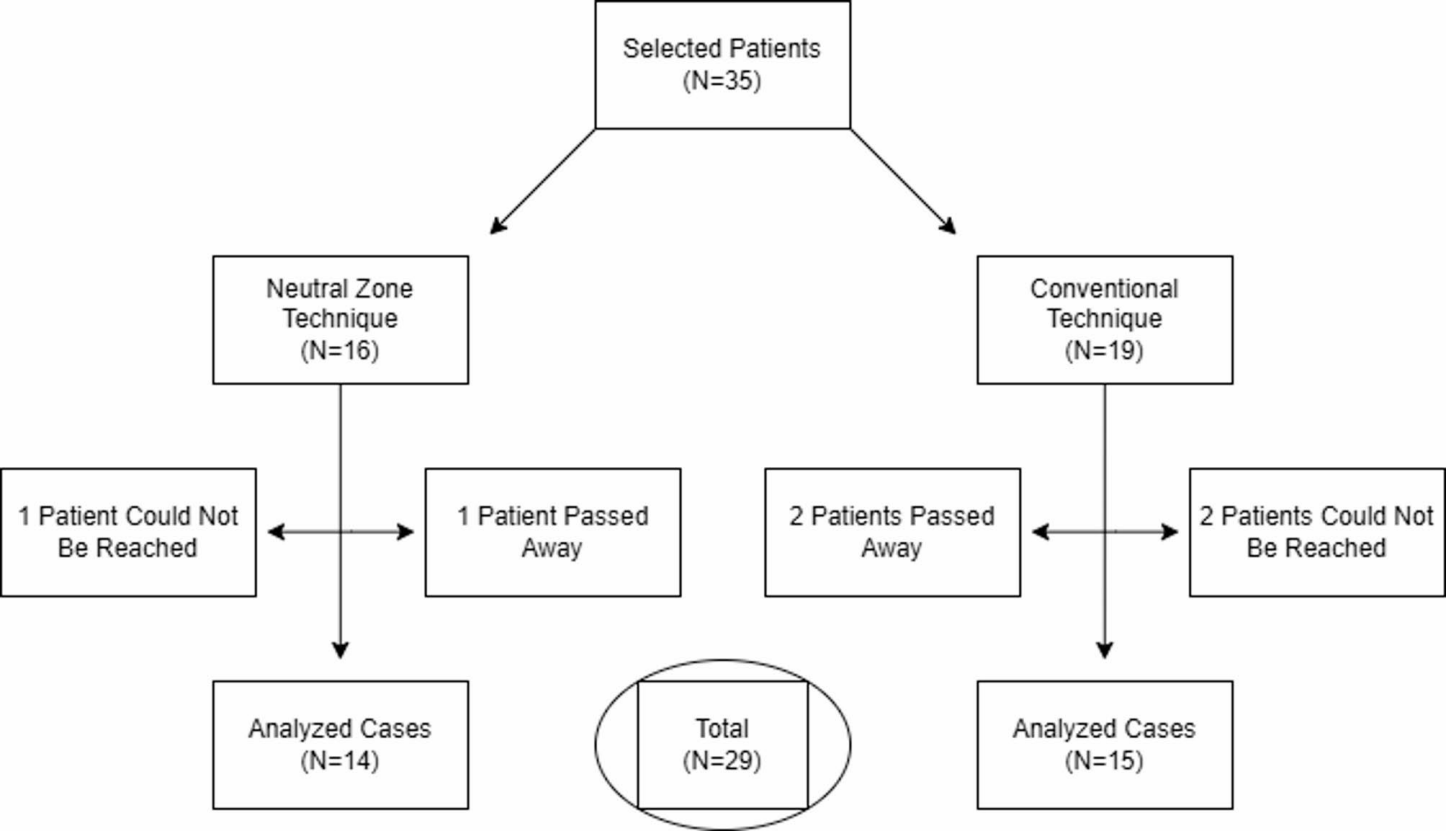
Flow diagram of the clinical study

After wearing each set of dentures for at least two years, patients completed the Turkish version of the OHIP-EDENT questionnaire to assess the dentures and their psychological impact on oral health. Participants rated how frequently their daily activities were affected by oral health issues related to denture use on a scale of 0–4 (0 = Never; 1 = Hardly ever; 2 = Sometimes; 3 = Fairly often; 4 = Very often). They were also asked about their pre-treatment status with OHIP-EDENT for comparative purposes. Due to potential bias in recalling circumstances from at least two years prior, those pretreatment data are not presented here. None of the patients who underwent both techniques experienced prosthesis loss. Furthermore, they did not need to completely replace their prostheses due to their own wishes, other than breakage.

### Statistical analysis

This study analyzed various parameters such as demographic and clinical characteristics, patient satisfaction, and functional and physical outcomes for differences between the Neutral Zone and Conventional groups. Descriptive statistics for continuous variables within the scope of the study were expressed by giving mean and standard deviation in case of compliance with normal distribution, and median and min-max values ​​in case of not provided. The normality assumption was tested with the Shapiro-Wilk test. For categorical variables, frequency or percentage values ​​were used while giving descriptive statistics. A paired design was used to test whether the paired difference (δ) in the distributions was different from 0 (H0: δ = 0 and H1: δ ≠ 0). The comparison was made using the two-sided, paired difference Wilcoxon Signed-Rank test with a Type I error rate (α) of 0.05. Based on data distribution, statistical analysis of group differences employed Student’s t-test or the Mann-Whitney U test for independent samples, based on data distribution. The Wilcoxon Signed Rank test was used for comparisons within groups. Fisher’s Exact Test was applied to examine relationships between categorical variables. A *p*-value < 0.05 was considered statistically significant. Clinical trial number: not applicable.

## Results

Figure 2 shows the patient flow and retention. Twenty-nine participants were included in the analysis: 14 in the Neutral Zone group and 15 in the Conventional group. The mean follow-up time was 51 ± 4.7 months for the Neutral Zone group and 49 ± 4.24 months for the Conventional group. The mean age of the participants was 64.28 ± 6.41 years (range 57–75 years). Fifteen participants were male, and fourteen were female.

No significant difference in mean age was found between the two groups (*p* > 0.05). Additionally, there was no significant difference in follow-up periods (*p* > 0.05). The distribution of males and females was similar across the groups (*p* > 0.05). Table 1 indicates that systemic disease or smoking status did not significantly differ between the groups (*p* > 0.05). No significant difference was observed concerning prosthesis fractures or the need for insertion of attachments (“locator”) replacement between groups (*p* = 1.000). Table 2 shows no significant difference in patient satisfaction prior to treatment between the two groups (*p* > 0.05). However, both groups experienced significant improvement after treatment (Neutral Zone: *p* < 0.001, conventional: *p* < 0.001). For functional assessment scores, a significant improvement was also noted in both groups (Neutral Zone: *p* < 0.001, Non-Neutral Zone: *p* < 0.001). Pre-treatment pain and physical disability scores revealed no significant difference between groups, but post-treatment improvement was statistically significant in both groups (*p* < 0.001). Similar improvements were observed in psychological discomfort and disability scores post-treatment (*p* < 0.001). Regarding social disability and handicap, no pre-treatment differences were noted between the two groups, but both groups showed significant improvement after treatment (*p* < 0.001). These findings indicate the effectiveness of both interventions, showing a comprehensive positive impact on patient outcomes. Post-treatment improvements in physical and psychological domains suggest a broad benefit of denture treatment in the study population.

**Table 1:** Demographic data distribution for neutral zone and conventional overdenture groups

Demographic	Neutral Zone(*n*:14)	Conventional(*n*:15)	Total (*n* = 29)	*p*
Age (Mean ± SD)	64,7143 ± 6,39024	63,8667 ± 6,62103	64,2759 ± 6,40812	0,729^a^
Follow Up (months)	60 (24–61)	58 (24–65)	60 (24–65)	0,847^b^
Gender				
Male	8 (57,1)	7 (46,7)	15 (51,7)	0,715^c^
Female	6 (42,9)	8 (53,3)	14 (48,3)	
Systematic Disease				
No	9 (64,3)	10 (66,7)	19 (65,5)	1,000^c^
Yes	5 (35,7)	5 (33,3)	10 (34,5)	
Smoking				
No	11 (78,6)	11 (73,3)	22 (75,9)	1,000^c^
Yes	3 (21,4)	4 (26,7)	7 (24,1)	
Prothesis Fracture				
No	12 (85,7)	12 (80,0)	24 (82,8)	1,000^c^
Yes	2 (14,3)	3 (20,0)	5 (17,2)	
Attachment Replacement				
No	7 (50,0)	8 (53,3)	15 (51,7)	1,000^c^
Yes	7 (50,0)	7 (46,7)	14 (48,3)	

a: Student t Test (Data presented as Mean ± St. Dev.)

b: Mann Whitney U Test (Data presented as Median (Min-Max))

c: Fisher’s Exact Test (Data presented as n (%))

**Table 2:** Patients’ satisfaction according to oral health impact profile for edentulous subjects (OHIP-EDENT)

Parameter	Neutral Zone	Conventional	*p* ^a^
Patient Satisfaction			
Before	49 (44–52)	48 (40–56)	0,715
After	12,5 (9–16)	14 (9–18)	0,234
p^b^	< 0,001	< 0,001	
Functional			
Before	9 (7–11)	9 (7–11)	0,813
After	2 (2–4)	3 (1–3)	0,914
p^b^	< 0,001	< 0,001	
Physical Pain			
Before	9 (6–11)	9 (7–11)	0,780
After	2 (1–4)	3 (2–4)	0,172
p^b^	< 0,001	< 0,001	
Psychological Discomfort			
Before	7 (5–11)	8 (5–11)	0,533
After	2 (1–3)	2 (1–3)	0,813
p^b^	< 0,001	< 0,001	
Physical Disability			
Before	9 (7–11)	9 (7–11)	0,983
After	2 (1–3)	2 (1–3)	0,652
p^b^	< 0,001	< 0,001	
Psychological Disability			
Before	6 (4–7)	6 (4–8)	0,683
After	2 (1–3)	2 (1–3)	0,158
p^b^	< 0,001	< 0,001	
Social Disability			
Before	5 (3–6)	5 (2–6)	0,747
After	1 (0–2)	1 (0–3)	0,158
p^b^	< 0,001	< 0,001	
Handicap			
Before	4 (2–5)	4 (2–6)	0,780
After	0,5 (0–1)	1 (0–2)	0,451
p^b^	< 0,001	< 0,001	

a: Mann Whitney U Test

b: Wilcoxon Signed Rank Test

Data presented as Median (Min-Max)

Two of the patients who underwent neutral zone had prosthesis fractures, which occurred at 23 and 36 months after the procedure. In the conventional group, prosthesis fractures occurred in 3 patients, which occurred at 13, 24 and 48 months, respectively. Attachment replacement occurred in 7 patients in the NZ group, with a median of 24 months (range 13–52 months), and in the CP group, attachment replacement was performed in 7 patients, with a median of 16 months (range 11–48 months). Wilcoxon Signed-Rank and Mann-Whitney U tests were calculated and presented in a Table 3.

**Table 3:** Mann-Whitney U and Wilcoxon tests for effect sizes

Tests Groups	Z	Effect Size	Interpretation
		(*|r|* = *|Z|*/*√N)*	
**Wilcoxon Tests– (Before - After Groups)**			
Patient Satisfaction Score Post Procedure - Patient Satisfaction Score Pre Procedure	-4.707	0.87	Large Effect Size
Post-functional– Pre-functional	-4.732	0.88	Large Effect Size
After physical pain– Before physical pain	-4.733	0.88	Large Effect Size
After psychological discomfort - Before psychological discomfort	-4.740	0.88	Large Effect Size
After physical disability - Before physical disability	-4.731	0.88	Large Effect Size
After psychological disability - Before psychological disability	-4.743	0.88	Large Effect Size
After social disability– Before social disability	-4.739	0.88	Large Effect Size
After handicap– Before handicap	-4.742	0.88	Large Effect Size
**Mann-Whitney U Tests– (Neutral Zone - Non-Neutral Zone Groups)**			
Time elapsed since procedure performed months	-0.226	0.04	Small Effect Size
Patient satisfaction score before procedure	-0.375	0.07	Small Effect Size
Patient satisfaction score after procedure	-1.245	0.23	Medium Effect Size
Pre-functional	-0.270	0.05	Small Effect Size
Post-functional	-0.142	0.03	Small Effect Size
Pre-physical pain	-0.293	0.05	Small Effect Size
Post-physical pain	-1.518	0.28	Medium Effect Size
Pre-psychological discomfort	-0.642	0.12	Small Effect Size
Post-psychological discomfort	-0.292	0.05	Small Effect Size
Pre-physical disability	-0.022	0.00	Negligible Effect Size
Post-physical disability	-0.543	0.10	Small Effect Size
Before psychological disability	-0.462	0.09	Small Effect Size
After psychological disability	-1.578	0.29	Medium Effect Size
Before social disability	-0.372	0.07	Small Effect Size
After social disability	-1.516	0.28	Medium Effect Size
Before handicap	-0.298	0.06	Small Effect Size
After handicap	-0.855	0.16	Small Effect Size

Note: Effect size (*r*) interpretation: *r* = 0.10 Small Effect Size; *r* = 0.30 Medium Effect Size; *r* = 0.50 Large Effect Size. *N* represents the number of pairs for Wilcoxon tests and the total number of participants for Mann-Whitney U tests. ”Negligible Effect Size” is considered for *r <* 0.10 (e.g., *r ≈* 0.00)

## Discussion

Any prosthetic dental treatment aims to provide a durable, problem-free prosthesis that is functionally satisfactory while preserving the health of supporting and surrounding oral structures. Accordingly, factors that may affect the long-term osseointegration of implants supporting and retaining mandibular overdentures and thus the health of the peri-implant tissues warrant investigation. In this study, we used OHIP-EDENT to assess whether denture stability, enhanced by the neutral zone technique, might influence patient satisfaction over the long term. No significant difference in satisfaction was observed between the two groups; therefore, the first hypothesis was rejected. However, reporting on the clinical applicability of the neutral zone approach with long-term follow-up is valuable, considering the limited literature on the topic. According to the results, there was no significant difference between the groups regarding prosthesis failure or attachment replacement, therefore, the second hypothesis was also rejected.

Multiple factors may account for the improvement in OHIP-EDENT scores in both groups. All patients presented to the clinic because they were dissatisfied with their existing dental condition, leading to high baseline impact scores. They all received their preferred treatment, and patient preference has been shown to influence satisfaction.^14^, ^18^]. Satisfaction is relative and is linked to treatment acceptance [19]. Patients who agreed to receive care in a dental school setting from a senior staff member rather than from undergraduate students might have perceived the care quality as superior. Such powerful contextual influences could have masked any potential outcome differences between the two denture fabrication techniques.

Despite the anticipated benefits of the neutral zone technique for complete denture function and stability, Rehmann et al [8]. concluded that the procedure could not be routinely recommended because of its relatively complex clinical steps, which require a skilled practitioner and a cooperative patient. Darwish et al. [9] compared bone loss around implants in overdenture patients treated with either the neutral zone or the conventional technique, finding no statistically significant differences. Although Darwish similarly hesitated to fully endorse the neutral zone method, the reasons were not explicitly stated. The lack of statistically significant superiority in both studies might reflect technical complexity and limited familiarity among clinicians and laboratory technicians [8, 9]. In contrast, Fahmy et al. [7] reported that while conventional complete dentures were superior in terms of mastication, neutral zone dentures provided better comfort and speech performance. However, their study offered only short-term (two week) follow-up results. In another study, significant improvements were also noted in masticatory function with the neutral zone technique in a small sample of five patients who could not undergo implant placement [8]. The present study found no statistically significant differences between the two groups regarding chewing function.

Al-Magaleh et al. [20] noted that the neutral zone (NZ) technique outperformed the conventional (CV) approach across all functional dimensions in edentulous patients without implant support. The reported superiority of NZ dentures in specific studies could be attributed to subjective perceptions that NZ dentures offer enhanced stability, retention, and comfort, accompanied by fewer post-insertion concerns [6, 7, 16, 21]. Al-Magaleh et al. further explained that such comfort stems from designing denture flanges to adapt to the patient’s tongue, lips, and cheeks at rest and during function, thereby improving denture stability and retention. However, in the present study, no statistically significant difference in functional scores was observed between the two groups.

Geerts et al. [10] used the OHIP-20 in a sample of edentulous patients without implants to compare the neutral zone with conventional denture techniques. Eight weeks after treatment, the hypothesized superiority of the NZ technique was not supported; the difference between NZ and conventional dentures was statistically insignificant. Similar to our findings, both techniques significantly improved patients’ quality of life from their pretreatment state. Our study also revealed statistically significant improvements in OHIP-EDENT scores post-treatment in both groups, with neither group demonstrating superiority in overall or subcategory scores.

According to previous studies, the maintenance cost of implant-retained overdentures is significantly affected by attachment systems, [22, 23, 24] with the recently introduced new attachment system, even providing promising results [25]. However, the location of the overdenture (maxilla versus mandibula) presented no significant difference in maintenance frequency [25]. The maintenance frequency and wear resistance data on the NZ technique are lacking. Although these techniques are designed to mitigate stress on overdentures while enhancing their functionality, the findings of this study indicate that no significant differences were observed between the two methods concerning maintenance frequency and wear resistance. It is important to acknowledge the multifactorial nature of maintenance frequency and wear resistance when interpreting these results. Furthermore, addressing the incorporation of oral hygiene maintenance and regular inspections of the overdentures is essential to minimize maintenance frequency effectively [26].

Patient satisfaction with dentures is multifactorial, influenced by technical aspects, dentist patient interactions, and patient specific factors such as age, gender, education level, compliance, and prior denture experience [26, 27, 28]. To reduce the influence of confounding variables, we designed this study to ensure that the same operator and laboratory technician fabricated all dentures, and we applied the same inclusion criteria to both techniques. Although we did not employ a crossover design (where each participant would receive both types of dentures sequentially), our methods minimized potential bias by maintaining consistency in clinical and laboratory protocols across both groups. Additionally, objective functional assessments such as bite force, masticatory efficiency, or speech analysis were not included, which could have provided a more comprehensive evaluation of the prostheses. Another limitation of our study is that the number of patients and clinicians are not higher and the clinicians cannot be compared and evaluated. Since our study is not a prospective study, the pre-satisfaction survey was conducted later. This is another limitation of our study. Despite these limitations, the study offers valuable insights into the long-term impact of conventional and neutral zone implant-retained complete dentures on oral health-related quality of life.

## Conclusion

The findings of this study indicate that implant supported overdenture prostheses fabricated using the neutral zone technique do not offer a long-term advantage over conventionally fabricated prostheses in terms of patient satisfaction, oral health related quality of life and prosthodontic maintenance. Nevertheless, both prosthetic approaches provided notable benefits in terms of functional improvement, discomfort reduction, and the maintenance of healthy peri-implant tissues. The results underscore that individualized treatment planning and interdisciplinary evaluations are fundamental for enhancing clinical success and sustaining patient satisfaction. In the future, multicenter prospective studies with larger sample sizes will be crucial in clarifying the potential advantages of the neutral zone technique, thereby refining its application protocols and indications.

## Data Availability

The data that support the findings of this study are available from the corresponding author upon reasonable request.

## Abbreviations

CD: Complete dentures
CV: Conventional (conventional technique/method)
NZ: Neutral zone
OHIP: Oral health impact profile
OHIP-14: Oral health impact profile-14
OHIP-EDENT: Oral health impact profile for edentulous subjects
OHRQoL: Oral health–related Quality of Life
PBOs: Patient-based outcomes (patient-centered measures)
